# m^6^A epitranscriptomic remodeling links redox stress to mitochondrial quality control and programmed cell death in sepsis-induced myocardial dysfunction

**DOI:** 10.1016/j.redox.2026.104178

**Published:** 2026-04-19

**Authors:** Meilian Chen, Binlan Fu, Qiaomin Wu

**Affiliations:** a Cardiac and Pulmonary Department, Quanzhou Hospital of Traditional Chinese Medicine, Fujian, 362000, China; b Department of Internal Medicine, Chen Dai Central Health Center, Jinjiang, 362256, China; c Department of Cardiology, Guang'anmen Hospital, China Academy of Chinese Medical Sciences, Beijing, 100053, China

**Keywords:** m6A, Oxidative stress, Mitochondria, Pyroptosis, Ferroptosis, Sepsis

## Abstract

Sepsis-induced myocardial dysfunction (SIMD) is a major contributor to sepsis-related mortality and is characterized by excessive oxidative stress, mitochondrial dysfunction, and heterogeneous forms of programmed cell death. However, how cardiomyocytes interpret redox stress and commit to distinct death pathways remains incompletely understood. Increasing evidence suggests that N^6^-methyladenosine (m^6^A), the most abundant internal RNA modification, functions as a dynamic post-transcriptional regulator linking redox signaling to mitochondrial homeostasis and cell fate decisions.

This review summarizes recent advances indicating that m^6^A-dependent regulatory networks integrate mitochondrial reactive oxygen species (mtROS), mitochondrial quality control (MQC), and downstream cell death pathways in SIMD. Under septic conditions, sustained inflammation and oxidative stress perturb the balance of m^6^A writers, erasers, and readers, leading to maladaptive remodeling of mitochondrial dynamics, mitophagy, and biogenesis. Such epitranscriptomic dysregulation is associated with mtROS accumulation, impaired mitochondrial renewal, and a shift from adaptive redox compensation toward irreversible cardiomyocyte injury.

Importantly, emerging evidence suggests that m^6^A remodeling does not uniformly activate cell death but modulates redox signal processing in a context-dependent manner. Preferential amplification of inflammatory sensing and inflammasome signaling may bias mtROS toward pyroptotic execution, whereas compromised antioxidant capacity, iron handling, and lipid metabolism may increase vulnerability to ferroptosis. On this basis, we propose the m^6^A–ROS–MQC axis as a unifying, hypothesis-driven framework for understanding SIMD pathogenesis, in which m^6^A acts as a redox-responsive epitranscriptomic regulator coordinating mitochondrial adaptation and programmed cell death decisions.

## Highlights

• m^6^A links redox stress to mitochondrial quality control in septic hearts.
• Dysregulated m^6^A amplifies mtROS and disrupts mitochondrial homeostasis.
• m^6^A biases redox signaling toward pyroptosis or ferroptosis.
• m^6^A acts as an epitranscriptomic decision node in SIMD.
• Targeting m^6^A–mitochondrial crosstalk offers therapeutic potential.

## 1 Introduction

Sepsis-induced myocardial dysfunction (SIMD) represents one of the most lethal complications of sepsis and is characterized by acute onset, complex pathophysiology, and profound adverse effects on both short- and long-term outcomes [1,2]. Clinically, myocardial dysfunction occurs in approximately 40–60% of patients with severe sepsis or septic shock and is independently associated with increased mortality, prolonged intensive care unit (ICU) stay, and a higher risk of multi-organ failure [3]. Despite advances in early goal-directed therapy, antimicrobial strategies, and supportive critical care, no specific cardioprotective therapy has been established for SIMD. Current management remains largely supportive, relying on fluid resuscitation, vasopressors, and inotropes, which often provide only transient hemodynamic stabilizationwhile failing to address the underlying molecular drivers of myocardial injury.

At its pathological core, SIMD is driven by redox imbalance–induced mitochondrial dysfunction, leading to collapse of myocardial energy metabolism and amplification of programmed cell death cascades [4]. Excessive reactive oxygen species (ROS) act as both damaging agents and signaling mediators, priming mitochondrial dysfunction and lowering the activation threshold of multiple regulated cell death pathways, including pyroptosis and ferroptosis [5]. Importantly, the reversible nature of SIMD observed in some survivors suggests that myocardial injury during sepsis is not purely structural but involves dynamic regulatory networks that determine whether cardiomyocytes undergo recovery or irreversible death [6]. However, the molecular determinants that govern this critical transition remain incompletely understood. Although anti-inflammatory and antioxidant strategies can partially improve cardiac performance [7,8], their limited clinical efficacy suggests that targeting ROS alone is insufficient to disrupt the self-amplifying pathogenic cycle of SIMD.

Recent advances in epitranscriptomics have provided a new perspective for understanding cellular adaptation to oxidative stress. N^6^-methyladenosine (m^6^A), the most abundant and reversible internal modification of eukaryotic mRNA, exhibits dynamic and context-dependent regulation, enabling rapid remodeling of gene expression programs in response to environmental stressors [9]. Accumulating evidence from models of ischemia–reperfusion injury, heart failure, and inflammatory diseases indicates that m^6^A modification critically regulates metabolic homeostasis, apoptosis, and immune responses, thereby shaping cardiomyocyte stress tolerance and functional remodeling. Importantly, m^6^A regulation extends beyond transcriptional fine-tuning and directly influences MQC, redox signaling, and downstream cell fate decisions, positioning it as a key determinant of cellular energy balance and survival under oxidative stress conditions. Beyond experimental models, emerging evidence from human sepsis cohorts further supports the clinical relevance of m^6^A dysregulation. Blood-based transcriptomic analyses have revealed widespread alterations in m^6^A regulators, including methyltransferase-like 3 (METTL3), fat mass and obesity-associated protein (FTO), and AlkB homolog 5, RNA demethylase (ALKBH5), which are associated with immune activation and inflammatory burden in septic patients [10]. However, cardiac-specific human evidence linking m^6^A regulation to myocardial mitochondrial dysfunction or redox imbalance remains scarce, underscoring a critical translational gap.

Given that sepsis is a highly dynamic and systemic inflammatory syndrome characterized by abrupt redox shifts, metabolic reprogramming, and heterogeneous cell death responses, a regulatory system capable of rapidly integrating environmental stress signals and reprogramming transcript fate is likely to play a decisive role in myocardial adaptation or failure. In this context, m^6^A methylation emerges as a redox-sensitive epitranscriptomic regulator linking oxidative stress, mitochondrial dysfunction, and divergent programmed cell death pathways. Understanding how m^6^A-dependent epitranscriptomic remodeling contributes to SIMD may therefore uncover novel mechanistic insights and identify precision therapeutic targets beyond conventional anti-inflammatory or antioxidant approaches.

**Fig. 1:**
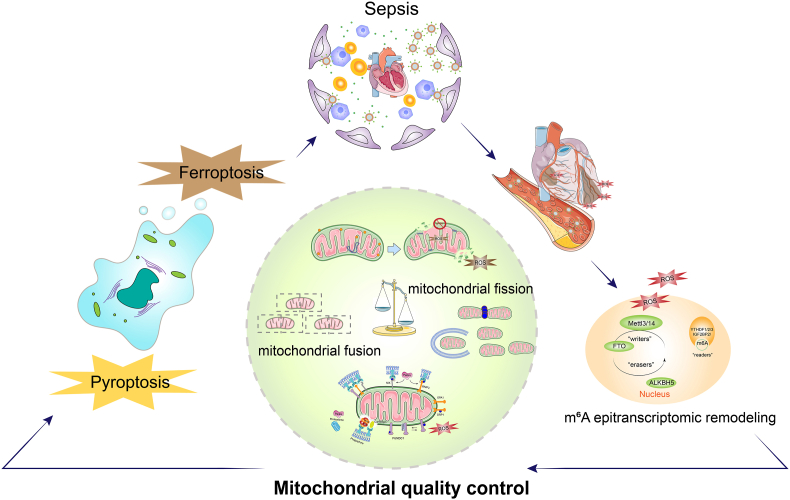
m^6^A–ROS–mitochondrial quality control axis orchestrates cell fate decisions in sepsis-induced myocardial dysfunction Sepsis triggers a systemic inflammatory response characterized by excessive cytokine release and oxidative stress, which converges on the heart and contributes to myocardial dysfunction. Under septic conditions, sustained ROS production is associated with epitranscriptomic remodeling mediated by dysregulated m^6^A machinery, including altered activity of m^6^A writers (METTL3/14), erasers (FTO, ALKBH5), and readers (YTHDF1/2/3, IGF2BP family). m^6^A-dependent post-transcriptional reprogramming is proposed to influence MQC by modulating mitochondrial dynamics (fusion and fission), mitophagy, and mitochondrial integrity. Imbalance between mitochondrial fusion and fission may lead to mitochondrial fragmentation, enhanced mitochondrial ROS generation, and bioenergetic failure. Accumulation of dysfunctional mitochondria further exacerbates oxidative stress, forming a feed-forward loop between ROS and MQC disruption. At the downstream level, m^6^A-associated redox signaling is hypothesized to bias cardiomyocyte fate decisions toward distinct forms of programmed cell death. Preferential amplification of inflammatory sensing and inflammasome activation may promote pyroptosis, whereas impaired antioxidant defense, iron homeostasis, and lipid redox balance predispose cardiomyocytes to ferroptosis.

Based on these observations, this review explicitly proposes the “m^6^A–ROS–MQC axis” as a core theoretical framework underlying the pathogenesis of SIMD (Fig. 1). We systematically examine how m^6^A-dependent epitranscriptomic remodeling integrates oxidative stress, mitochondrial dysfunction, and programmed cell death, and discuss its potential role in maintaining myocardial redox homeostasis and modulating cell fate decisions. By framing m^6^A as a redox-responsive regulatory hub rather than a passive RNA modification, this perspective aims to highlight its potential as a precision target for modulating mitochondrial function and interrupting pathogenic redox signaling in SIMD, thereby offering a hypothesis-driven theoretical foundation for epitranscriptome-based therapeutic strategies. To enhance the rigor and traceability of the evidence summarized in this review, we explicitly indicate the experimental context of cited findings—such as cell-based studies, animal models, or human clinical samples—when describing published results. This distinction is important because mechanistic insights derived from preclinical systems may not fully translate to the human septic myocardium.

## 2 Oxidative stress and mitochondrial dysfunction in sepsis-induced myocardial dysfunction

Oxidative stress represents both an initiating signal and a central driving force in the pathogenesis of SIMD. Under the hyperinflammatory milieu of sepsis, excessive activation of immune cells leads to aberrant stimulation of multiple ROS-generating systems, including nicotinamide adenine dinucleotide phosphate (NADPH) oxidase (NOX) complexes, xanthine oxidase (XO), and the mitochondrial electron transport chain (mETC), resulting in the overproduction of ROS and reactive nitrogen species (RNS) [11,12]. When ROS generation exceeds the cellular antioxidant capacity, key redox-buffering systems—such as the glutathione (GSH)–glutathione peroxidase (GPX) axis, the thioredoxin (Trx) system, and superoxide dismutases (SODs)—become progressively exhausted. This imbalance promotes lipid peroxidation, protein thiol oxidation, and oxidative DNA damage, ultimately disrupting redox homeostasis and placing cardiomyocytes under sustained oxidative stress [13].

Mitochondria function as both major sources and primary amplification hubs of oxidative stress during sepsis. In response to pathogen-associated molecular patterns (PAMPs) and damage-associated molecular patterns (DAMPs), pattern recognition receptors such as Toll-like receptors (TLRs) and NOD-like receptors (NLRs) activate nuclear factor kappa-B(NF-κB) and MAPK signaling pathways. These signals converge on the mETC, promoting electron leakage from complexes I and III and accelerating the generation of mitochondrial ROS (mtROS) [14,15]. Beyond causing direct oxidative damage to mitochondrial lipids—such as cardiolipin—and respiratory chain proteins, mtROS also function as critical signaling mediators that amplify inflammatory and stress-response pathways [16]. In turn, excessive mtROS establish a self-amplifying pathogenic loop in which mitochondrial dysfunction and oxidative stress reinforce each other, progressively sensitizing cardiomyocytes to inflammatory injury and regulated cell death.

The MQC system constitutes a critical safeguard for maintaining mitochondrial integrity and functional homeostasis. During sepsis, however, excessive ROS and inflammatory signaling globally disrupt MQC coordination, resulting in impaired removal of damaged mitochondria and insufficient mitochondrial renewal [17]. As a result, dysfunctional mitochondria accumulate, mtROS production becomes sustained, and mitochondrial damage is progressively amplified, establishing a vicious cycle that drives persistent oxidative stress and myocardial injury in SIMD [[18], [19], [20], [21]].

Beyond direct structural damage, oxidative stress profoundly perturbs myocardial energy metabolism in sepsis. Inflammatory mediators and excessive nitric oxide (NO) suppress mitochondrial oxidative phosphorylation (OXPHOS), forcing cardiomyocytes to shift toward glycolysis as the dominant adenosine triphosphate (ATP)-producing pathway [22]. Although this metabolic reprogramming may transiently sustain energy production, it is accompanied by lactate accumulation, disruption of the NAD^+^/NADH redox balance, and inhibition of pyruvate dehydrogenase (PDH), thereby exacerbating mitochondrial membrane depolarization and ROS leakage [23]. Simultaneously, impaired fatty acid oxidation further compromises myocardial energy supply, deepening the energetic deficit [24].

This bioenergetic failure reinforces oxidative stress by limiting the regeneration of essential antioxidant systems, rendering cardiomyocytes increasingly vulnerable to redox injury. Consequently, multiple programmed cell death pathways—such as apoptosis, pyroptosis, and ferroptosis—are coordinately activated, accelerating cardiomyocyte loss and contractile dysfunction.

Taken together, oxidative stress and mitochondrial dysfunction form a self-amplifying pathogenic loop in SIMD, in which excessive ROS production initiates mitochondrial impairment, while mitochondrial dysfunction further enhances ROS release. This oxidative stress–mitochondria–cell death axis represents a central molecular framework underlying SIMD progression and provides a critical mechanistic foundation for subsequent discussion of how m^6^A epitranscriptomic regulation intersects with redox signaling and mitochondrial quality control.

## 3 m^6^A methylation as a redox-sensitive epitranscriptomic switch

As a redox-sensitive epitranscriptomic switch, we propose that m^6^A modification undergoes stage-specific reprogramming during sepsis progression. Moderate elevation of ROS in the early stage upregulates METTL3, triggering adaptive m^6^A remodeling and supporting reversible cardiomyocyte adaptation. In contrast, excessive ROS accumulation in the sustained injury phase leads to dysregulation of core m^6^A regulators (e.g., METTL3 and FTO), which promotes aberrant methylation of transcripts involved in mitophagy, antioxidant defense, and pyroptosis, ultimately contributing to irreversible cardiomyocyte death. In the late repair phase, the restoration of redox homeostasis rebalances the m^6^A regulatory machinery to facilitate tissue repair and recovery. Although direct temporal evidence in septic myocardium remains limited, m^6^A methylation is tightly synchronized with oxidative stress dynamics and cardiomyocyte fate transition.

### 3.1 Redox-driven reprogramming of the m^6^A regulatory machinery

Oxidative stress represents the central pathological driver of SIMD. In this context, ROS function not only as effectors of cellular damage but also as critical signaling inputs that dynamically reshape post-transcriptional regulatory systems. Through convergent redox-sensitive signaling pathways, ROS coordinately modulate the expression, activity, and functional balance of m^6^A writers, erasers, and readers, thereby reprogramming RNA fate decisions that govern cardiomyocyte adaptation, dysfunction, or death under septic stress.

#### 3.1.1 Writers: redox-driven signal amplification

During sepsis, transient lactate accumulation reflects mitochondrial stress and contributes to oxidative injury, promoting mitochondria-dependent ferroptosis [25]. In experimental inflammatory or metabolic stress models, lactate has been reported to enhances methyltransferase-like 3 (METTL3) transcription by increasing p300-mediated histone H3K18 lactylation at the METTL3 promoter, thereby amplifying inflammatory damage [25]. In endothelial cells, ageing mouse models, and human vascular samples, the m^6^A methyltransferase-like 14 (METTL14) selectively recognizes and binds the 3′ untranslated region (3′UTR) of toll-like receptor 4 (TLR4) mRNA, increasing its m^6^A modification. This modification significantly stabilizes TLR4 transcripts, resulting in elevated TLR4 protein expression [26]. Enhanced TLR4 signaling subsequently activates the canonical MyD88–NF-κB inflammatory cascade, triggering a senescence-associated secretory phenotype (SASP)-like response characterized by excessive production of cytokines, chemokines, growth factors, and ROS, thereby reinforcing redox imbalance [26].

Beyond transcriptional regulation, ROS also modulate m^6^A writer activity through post-translational mechanisms. In cellular models, oxidative stress–activated ERK and JNK signaling promotes post-translational modification of ALKBH5, indirectly shifting the global balance toward increased m^6^A methylation as part of the cellular response to ROS-associated stress signaling [27]. Thus, redox cues can upregulate writer-driven methylation programs via both transcriptional (e.g., METTL3 induction) and pathway-coupled regulatory modes, thereby amplifying stress-responsive inflammatory outputs.

#### 3.1.2 Erasers: redox-dependent loss of reversibility

The m^6^A demethylases FTO and ALKBH5 exhibit pronounced sensitivity to cellular redox status. FTO requires Fe^2+^ and α-ketoglutarate (α-KG) as cofactors for its oxidative demethylation activity, as demonstrated in biochemical and cellular studies [28]. Under oxidative stress, Under oxidative stress, redox imbalance and metabolic perturbations have been shown in experimental models to compromise FTO enzymatic activity, resulting in reduced demethylase function and a global increase in m^6^A methylation [29,30]. While this shift may transiently facilitate adaptive translation of stress-responsive transcripts, sustained FTO inhibition ultimately disrupts epitranscriptomic reversibility and post-transcriptional homeostasis, thereby predisposing cells to maladaptive stress responses.

In contrast, ALKBH5 plays a context-dependent protective role in mitigating ROS-induced DNA damage and apoptosis. In cellular hypoxia models, ALKBH5 expression is induced through hypoxia inducible factor 1 subunit alpha (HIF-1α)–dependent signaling [31]. However, in cellular models, ROS-mediated activation of ERK/JNK pathways promotes ALKBH5 SUMOylation, sterically limiting substrate accessibility and suppressing its m^6^A demethylase activity [27]. This functional inhibition of ALKBH5, together with compensatory METTL3 upregulation and recruitment to DNA damage–associated regions, further biases the epitranscriptomic landscape toward sustained methylation programs [27]. At the transcript-specific level, ALKBH5-dependent demethylation has been shown to regulate stress-responsive transcripts and immune cell homeostasis in septic animal and cellular models, underscoring the in vivo consequences of redox-driven disruption of m^6^A eraser function [32].

Ultimately, persistent oxidative stress suppresses m^6^A eraser activity at both enzymatic and regulatory levels, eroding the reversibility of RNA methylation and locking cells into a sustained stress-associated epitranscriptomic state.

#### 3.1.3 Readers: translating redox signals into fate decisions

m^6^A reader proteins constitute critical nodes through which ROS signaling is translated into downstream post-transcriptional effects. Under oxidative stress conditions, m^6^A-modified mRNAs preferentially accumulate within stress granules (SGs), where they participate in adaptive translational repression. In cellular stress models, YTHDF family proteins are essential for SG assembly and mRNA triage, as depletion of YTHDF1 or YTHDF3 markedly impairs SG formation and limits mRNA recruitment into these structures [33]. Through this mechanism, m^6^A readers contribute to cellular stress adaptation by dynamically reshaping mRNA localization and translational availability.

Beyond stress granule–mediated adaptation, m^6^A readers exert context-dependent control over inflammatory signaling and cell death pathways during sepsis. In septic cellular and animal models, YTHDF1 promotes expression of the E3 ubiquitin ligase WWP1, thereby facilitating ubiquitination of NOD-like receptor family pyrin domain containing 3 (NLRP3) and suppressing caspase-1 (CASP1)–dependent pyroptosis, which contributes to attenuation of septic injury [34]. In contrast, YTHDF2 expression is markedly reduced in septic conditions, including peripheral blood mononuclear cells from septic mice and patients and lipopolysaccharide (LPS)-stimulated macrophages [35]. Loss of YTHDF2 stabilizes pro-inflammatory mRNAs, such as interleukin-6 receptor (IL-6R), leading to enhanced JAK2/STAT1 signaling and increased release of high-mobility group box 1 (HMGB1) [35].

Notably, m^6^A readers also influence mitochondrial homeostasis, as in cellular stress and experimental models, YTHDF2 regulates the m^6^A-dependent turnover of mitochondria-related transcripts, thereby constraining ROS accumulation and preserving mitochondrial function under stress conditions [36].

Taken together, m^6^A reader proteins act as redox-sensitive effectors that convert oxidative stress–encoded RNA methylation patterns into coordinated post-transcriptional programs governing inflammation, mitochondrial integrity, and cell fate decisions. Rather than serving as passive mRNA binders, YTHDF proteins function as dynamic interpreters of redox context, biasing cellular responses toward adaptive remodeling or maladaptive injury.

### 3.2 m^6^A-dependent non-coding RNA networks in redox regulation and mitochondrial control

Non-coding RNAs (ncRNAs) constitute a critical amplification layer within the epitranscriptomic landscape, enabling m^6^A signals to be propagated and integrated across multiple transcripts and signaling pathways. Under oxidative stress conditions, dynamic m^6^A modifications on ncRNAs reshape their stability, subcellular localization, and interactions with RNA-binding proteins, thereby functionally coupling redox imbalance to mitochondrial dysfunction and cell fate decisions.

Although accumulating studies have identified m^6^A modification on lncRNAs, circRNAs, and primary miRNA transcripts using methylated RNA immunoprecipitation sequencing (MeRIP-seq) and m^6^A-seq approaches, precise single-nucleotide resolution mapping of m^6^A sites on many ncRNAs remains incomplete in current cellular and experimental studies [37]. In most cases, modification peaks have been detected within consensus RRACH motifs, but functional validation of specific methylated adenosines (e.g., by site-directed mutagenesis or miCLIP-based mapping) is still limited [38]. Therefore, current evidence primarily supports the presence of m^6^A enrichment regions rather than fully characterized site-specific modification landscapes for many redox-related ncRNAs.

#### 3.2.1 lncRNAs: m^6^A-modified hubs linking mitochondrial quality control and inflammatory amplification

Long non-coding RNAs (lncRNAs) represent prominent m^6^A-modified targets that participate in mitochondrial homeostasis through regulation of mitophagy, redox signaling, and inflammatory amplification. In cellular and experimental disease models, several m^6^A-dependent lncRNAs, including metastasis-associated lung adenocarcinoma transcript 1 (MALAT1) and nuclear paraspeckle assembly transcript 1(NEAT1), exhibit enhanced stability and have been shown to promote inflammatory signaling while exacerbating mitochondrial stress responses [[39], [40], [41], [42], [43]].

Mechanistically, in cellular models, METTL3-mediated m^6^A deposition and subsequent recognition by YTHDF family proteins markedly increase MALAT1 stability [44]. m^6^A enrichment sites within MALAT1 have been detected predominantly in its 3′ region; however, only a subset of these sites has undergone functional interrogation, and the exact adenosines responsible for redox-related phenotypes remain to be definitively validated [45]. Elevated MALAT1 levels are associated with mitochondrial membrane potential collapse, excessive ROS accumulation, and aggravated endothelial or cardiomyocyte injury in cellular and animal models [42]. Conversely, suppression of MALAT1 restores mitophagic flux and improves mitochondrial redox homeostasis [42], underscoring the context-dependent impact of m^6^A-modified lncRNAs on mitochondrial quality control.

Functionally, lncRNAs frequently act as scaffolding molecules or competing endogenous RNA (ceRNA) platforms that integrate m^6^A readers with redox-sensitive transcriptional circuits, such as NF-κB or NLRP3 signaling in inflammatory cellular models [46,47]. Through these hierarchical interactions, m^6^A-modified lncRNAs often amplify inflammatory–mitochondrial coupling and lower the activation threshold for pyroptotic cell death.

#### 3.2.2 circRNAs: m^6^A-enabled modulators of mitochondrial stress responses

Circular RNAs (circRNAs), characterized by their covalently closed loop structures, exhibit enhanced stability and constitute a stress-resilient RNA reservoir under oxidative conditions. Emerging evidence indicates that m^6^A modification regulates circRNA turnover, translational potential, and interactions with RNA-binding proteins, thereby influencing mitochondrial function and redox signal transduction in cellular and experimental disease models [48].

Unlike linear RNAs, circRNAs may harbor internal m^6^A sites that enable cap-independent translation through recruitment of YTHDF3 or eIF4G2 in cellular models [49]. Although m^6^A peaks have been identified on multiple stress-responsive circRNAs, comprehensive base-resolution mapping of functional methylation sites in mitochondrial or ferroptosis-related circRNAs remains scarce.

Functionally, multiple circRNAs have been implicated in the regulation of mitochondrial DNA stability, ROS accumulation, and programmed cell death in cellular and animal models. Certain circRNAs exacerbate mitochondrial oxidative injury through poly ADP-ribose polymerase 1 (PARP1)/HMGB1-related pathways [[50], [51], [52]], whereas others appear to buffer redox stress by acting as miRNA sponges or by facilitating adaptive translation under stress conditions. This duality suggests that circRNAs may exert either synergistic or compensatory effects relative to lncRNA-mediated inflammatory amplification, depending on the directionality of m^6^A remodeling and reader engagement.

#### 3.2.3 miRNAs: m^6^A-dependent fine-tuners of redox and mitochondrial signaling

MicroRNAs (miRNAs) provide an additional layer of post-transcriptional fine-tuning for genes involved in mitochondrial function and redox homeostasis. Accumulating evidence demonstrates that m^6^A modification influences miRNA biogenesis and maturation, thereby indirectly shaping mitochondrial integrity and oxidative stress responses in cellular and experimental models [53,54]. In contrast to lncRNAs and circRNAs, m^6^A sites on pri-miRNAs have been more clearly characterized in several models, with defined methylated adenosines enhancing Microprocessor complex recruitment [53]. Nevertheless, for many redox- or mitochondrial quality control–related miRNAs in sepsis models, direct mapping of functional m^6^A sites remains limited.

Through modulation of key signaling pathways—including TLR4/NF-κB, PI3K/AKT, and MAPK cascades—miRNAs regulate the production of inflammatory mediators such as tumor necrosis factor-α (TNF-α), interleukin-6 (IL-6), and interleukin-1β (IL-1β), thereby influencing sepsis progression in cellular and animal models [55,56]. Importantly, miRNAs often counterbalance excessive inflammatory amplification driven by lncRNAs, functioning as buffering nodes that restrict the magnitude or duration of redox signaling [57]. Thus, whereas m^6^A-modified lncRNAs may preferentially promote pyroptotic or inflammatory phenotypes, m^6^A-dependent miRNAs can exhibit antagonistic effects by repressing pro-death transcripts.

#### 3.2.4 Synergistic and antagonistic interactions among m^6^A-modified ncRNAs

Rather than operating in isolation, lncRNAs, circRNAs, and miRNAs form an interconnected regulatory network in which m^6^A modification reshapes their hierarchical relationships. In cellular and experimental disease models, m^6^A-stabilized lncRNAs enhance inflammatory transcriptional programs and suppress mitophagy [58], thereby synergizing with mtROS accumulation to promote pyroptosis [59]. In contrast, specific miRNAs processed in an m^6^A-dependent manner may degrade transcripts encoding inflammasome components or ferroptosis mediators, exerting a compensatory or antagonistic effect in cellular models [60]. CircRNAs occupy an intermediate regulatory position: depending on their binding partners and reader engagement, they may either amplify inflammatory signaling (synergistic effect) or sequester pro-death miRNAs and mitigate mitochondrial injury (antagonistic effect) in cellular and animal models [61].

Therefore, different classes of ncRNAs do not exhibit uniformly cooperative behavior. Instead, m^6^A remodeling establishes a dynamic balance between amplification circuits (often lncRNA-dominant) and buffering circuits (often miRNA-mediated), with circRNAs functioning as modulatory hubs. The net outcome—mitochondrial adaptation versus death commitment—depends on the relative dominance of these opposing forces under specific redox conditions.

### 3.3 Redox–m^6^A feedback loops in cardiomyocyte stress adaptation

Although chronic cardiovascular diseases and acute SIMD differ markedly in temporal dynamics, they converge at the molecular level on a shared ROS–m^6^A–mitochondrial axis. As an energy-demanding cell type, cardiomyocytes rely on finely tuned redox signaling to adapt to metabolic and inflammatory stress. In this context, ROS function not only as damaging agents but also as signaling cues, whereas the m^6^A epitranscriptomic machinery acts as a post-transcriptional signal integrator, translating redox inputs into coordinated gene expression programs that determine cardiomyocyte adaptation or failure.

#### 3.3.1 Chronic stress phase: m^6^A-mediated adaptive remodeling under sustained redox pressure

In chronic cardiovascular conditions such as atherosclerosis, hypertension, and diabetes, cardiomyocytes are exposed to prolonged, moderate ROS stimulation. Under these conditions, adaptive activation of the m^6^A machinery contributes to the maintenance of metabolic homeostasis and stress tolerance [62]. Accumulating evidence from cardiovascular disease models highlights the pathological relevance of dynamic m^6^A regulation.

Dorn and colleagues demonstrated that in cardiac hypertrophy models, the m^6^A “writer” METTL3 promotes pathological hypertrophic remodeling via activation of MAPK signaling [9]. Adenoviral overexpression of METTL3 increased the expression of MAP3K6, MAP4K5, and MAPK14, while enhancing m^6^A methylation on hypertrophy-related transcripts such as Serca2a, Sox6, and Hsp70, thereby improving their translational efficiency and driving metabolic reprogramming and cardiomyocyte enlargement. Conversely, cardiomyocyte-specific deletion of METTL3 effectively blocked hypertrophic growth in vivo [9]. Notably, METTL3 overexpression induced compensatory hypertrophy without overt cardiac dysfunction, whereas long-term METTL3 deficiency led to progressive structural abnormalities and heart failure–like phenotypes in mouse models, underscoring the essential role of m^6^A in maintaining cardiac homeostasis and stress responsiveness [9].

In contrast, the m^6^A “eraser” FTO exerts cardioprotective effects under ischemic stress. During ischemia–reperfusion injury, FTO stabilizes transcripts encoding anti-apoptotic and calcium-handling proteins such as BCL2 and SERCA2a, thereby reducing cardiomyocyte apoptosis in cellular and animal ischemia–reperfusion models [63]. Consistently, downregulation of FTO in heart failure is associated with elevated global m^6^A levels, impaired contractility, and disrupted calcium homeostasis in heart failure animal models and human cardiac samples [64]. ALKBH5 has also been implicated in cardiac regeneration. In myocardial infarction animal models, ALKBH5 upregulation reduces m^6^A modification on YTHDF1 mRNA, enhancing its stability and indirectly promoting translation of the regenerative regulator YAP, which drives cardiomyocyte cell-cycle re-entry, limits scar formation, and improves cardiac function [65].

m^6^A reader proteins further refine stress adaptation. YTHDF2, a key mediator of m^6^A-dependent RNA decay [66], has been shown to attenuate hypoxia/reoxygenation-induced injury in cardiomyocyte cellular models by targeting BCL2 interacting protein 3 (BNIP3) mRNA for degradation, thereby alleviating mitochondrial dysfunction and apoptosis [67]. In parallel, YTHDF2-mediated recognition of mitsugumin 53 (MG53) mRNA enhances its stability, reducing ischemia–reperfusion–induced cardiomyocyte apoptosis in vivo in animal ischemia–reperfusion models in vivo [68]. Collectively, these findings position m^6^A regulators as central metabolic–transcriptional hubs that support adaptive cardiac remodeling under chronic redox stress.

#### 3.3.2 Acute stress phase: collapse of m^6^A homeostasis under overwhelming inflammatory ROS

In contrast to chronic adaptation, acute systemic inflammation during sepsis generates a rapid and excessive ROS surge that exceeds the buffering capacity of the m^6^A regulatory system. Transcriptome-wide analyses using MeRIP-seq and RNA-seq have revealed a pronounced increase in global m^6^A levels and METTL3 expression in the myocardium of LPS-induced sepsis models [69]. Mechanistically, METTL3-mediated m^6^A modification enhances histone deacetylase 4 (HDAC4) mRNA stability via the reader protein insulin like growth factor 2 mRNA binding protein 1 (IGF2BP1), thereby accelerating inflammatory injury in cardiomyocytes in cardiomyocyte cellular models [70]. Similarly, METTL14 has been shown to elevate m^6^A levels and promote the secretion of pro-inflammatory cytokines, including TNF-α, IL-1β, interleukin-3 (IL-3), and IL-6, driving endothelial and myocardial inflammation in cellular and animal inflammatory models [71].

Conversely, impaired m^6^A demethylation exacerbates septic cardiac dysfunction. In endotoxemia animal models and cardiomyocyte cellular experiments, reduced FTO expression in cardiomyocytes leads to elevated m^6^A levels and aberrant upregulation of inflammatory mediators such as IL-6, TNF-α, and interleukin-10 (IL-10), ultimately contributing to myocardial inflammation and contractile failure [72]. These observations support a model in which m^6^A dysregulation actively participates in sepsis progression by amplifying inflammatory signaling.

Beyond inflammation, m^6^A modifications directly influence regulated cell death pathways during SIMD. In LPS-treated H9C2 cardiomyocytes, METTL3-driven m^6^A modification promotes YTHDF2-dependent degradation of solute carrier family 7, membrane 11 (SLC7A11) mRNA, accelerating ferroptosis and cardiomyocyte apoptosis [73]. In contrast, in LPS-stimulated macrophages, acetyltransferase KAT2B stabilizes METTL14 via K398 acetylation, increasing m^6^A methylation on Spi2a transcripts and enhancing expression of this anti-inflammatory factor. This mechanism suppresses NF-κB signaling and indirectly mitigates sepsis-associated myocardial injury [74].

Taken together, these findings support a biphasic redox–m^6^A feedback model. Under physiological or moderate stress, m^6^A-mediated post-transcriptional regulation maintains redox and metabolic equilibrium, enabling mitochondrial adaptation and antioxidant defense. In contrast, under pathological and overwhelming stress, disruption of m^6^A homeostasis precipitates a collapse of post-transcriptional control, allowing ROS accumulation and mitochondrial dysfunction to mutually reinforce each other, thereby driving cardiomyocytes toward irreversible injury and death.

## 4 m^6^A-mediated programmed cell death

**Fig. 2:**
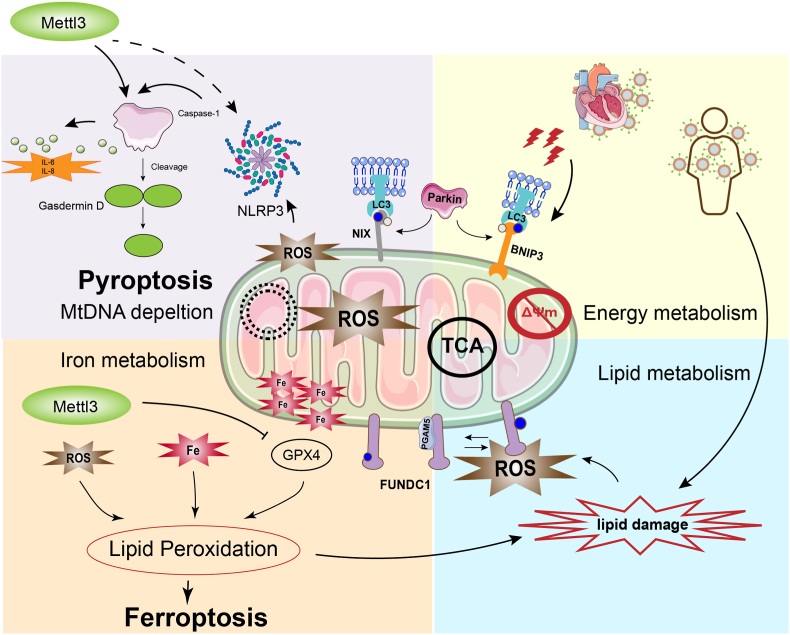
m^6^A-dependent integration of mitochondrial dysfunction, pyroptosis and ferroptosis in sepsis-induced myocardial injury This schematic illustrates a hypothesis-driven integrative model depicting how m^6^A epitranscriptomic remodeling may coordinate the convergence and divergence of mitochondrial dysfunction, pyroptosis, and ferroptosis under septic stress. In response to systemic inflammation and excessive ROS generation, mitochondrial quality control is disrupted, leading to impaired TCA cycle activity, loss of ΔΨm, and altered energy metabolism in cardiomyocytes. At the inflammatory axis, m^6^A remodeling has been implicated in facilitating NLRP3 inflammasome activation and CASP1-dependent cleavage of GSDMD, thereby contributing to pyroptotic cell death accompanied by cytokine release (e.g., IL-6 and IL-8) and mtDNA depletion. Mitochondrial damage further amplifies ROS production, reinforcing inflammasome signaling in a feed-forward manner. In parallel, m^6^A-associated dysregulation of iron and lipid metabolism is proposed to lower the threshold for ferroptosis by perturbing GPX4-dependent antioxidant defenses, promoting intracellular iron accumulation, and accelerating lipid peroxidation within mitochondrial membranes. Excessive lipid ROS generation ultimately drives ferroptotic execution, contributing to irreversible cardiomyocyte injury. Mitochondrial stress also activates compensatory mitophagy via LC3 recruitment and receptor-mediated pathways involving NIX and BNIP3, as well as PINK1–Parkin signaling. However, under persistent oxidative stress and sustained m^6^A imbalance, mitophagic clearance may become insufficient, allowing damaged mitochondria to accumulate and perpetuate ROS-driven cell death signaling.

In SIMD, cardiomyocyte death does not occur as a single or isolated biological event but rather represents a systems-level response arising from the convergence of oxidative stress, mitochondrial dysfunction, and epitranscriptomic regulation. Within this network, m^6^A methylation functions as a context-dependent epitranscriptomic hub, integrating redox-derived stress signals with multiple forms of programmed cell death, including pyroptosis, ferroptosis, and apoptosis/necroptosis-like pathways. This coordinated m^6^A–ROS–mitochondrial–cell death axis highlights the potential central role of m^6^A in translating inflammatory and metabolic stress into cell fate modulation, while dynamically modulating inflammatory amplification and myocardial injury severity (Fig. 2). Representative m^6^A regulators, their downstream targets, experimental models, and functional consequences relevant to SIMD are summarized in Table 1.

**Table 1:** Summary of representative m^6^A regulators and downstream targets in sepsis-induced myocardial dysfunction.

m^6^A regulator	Target transcript(s)	Model system	Major functional outcome	Level of evidence	Ref.
**METTL3** (writer)	**HDAC4**	LPS-induced SIMD mouse model; cardiomyocytes	Enhanced inflammatory signaling and myocardial dysfunction via m^6^A-IGF2BP1-HDAC4 axis	Animal, Cell	[70]
**METTL3** (writer)	**SLC7A11**	LPS-treated H9C2 cardiomyocytes	Impaired antioxidant defense, enhanced ferroptosis and apoptosis	Cell	[73]
**METTL3** (writer)	**ACSL4**	Sepsis-associated lung injury model	Promotion of lipid peroxidation and ferroptotic injury	Animal	[25]
**METTL3** (writer)	**pri-miR-193a → BCL2L2**	Cardiomyocytes under septic stress	Reduced anti-apoptotic capacity, mitochondrial instability	Cell	[75]
**METTL14** (writer)	**TLR4**	Endothelial cells and immune cells under septic stress	Stabilization of TLR4 mRNA and amplification of NF-κB signaling	Cell	[26]
**FTO** (eraser)	**PGC-1α**	LPS-treated cardiomyocytes; septic mouse heart	Preservation of mitochondrial biogenesis and energy metabolism	Cell, Animal	[76]
**FTO** (eraser)	**BNIP3/FUNDC1**	Cardiomyocytes under septic stress	Enhanced mitophagy and reduced mtROS accumulation	Cell	[77]
**YTHDF1** (reader)	**NLRP3** (via WWP1)	LPS-stimulated macrophages	Suppression of inflammasome activation and pyroptosis	Cell	[34]
**YTHDF2** (reader)	**IL-6R**	LPS-stimulated macrophages; septic mice	Attenuation of JAK2/STAT1 signaling and inflammatory response	Cell, Animal	[35]
**YTHDC1** (reader)	**Angptl4** (via lncRNA *Mir2*2hg)	Cardiomyocytes and metabolic stress models	Regulation of mitochondrial homeostasis and iron metabolism	Cell	[78]

### 4.1 m^6^A-regulated pyroptosis: a mitochondrial redox–driven inflammatory death pathway

Pyroptosis is a form of programmed inflammatory cell death characterized by inflammasome activation, inflammatory caspase cleavage, and gasdermin-mediated membrane pore formation. In SIMD, pyroptosis plays a pivotal role in inflammatory amplification and cardiac dysfunction. Accumulating evidence indicates that mitochondria-derived redox signals, particularly excessive mtROS, serve as critical upstream triggers that initiate and sustain pyroptotic signaling.

#### 4.1.1 m^6^A-dependent redox signaling lowers the activation threshold of pyroptosis

Under sepsis-associated oxidative stress, m^6^A modification influences the post-transcriptional fate of key inflammasome-related molecules, thereby shaping the sensitivity of cardiomyocytes and immune cells to pyroptotic activation. m^6^A “writers,” such as METTL3, have been shown to enhance the stability and/or translational efficiency of transcripts encoding core pyroptotic components, including NLRP3, apoptosis-associated speck -like protein containing a CARD (ASC), and CASP1, thereby potentially lowering the activation threshold of inflammasome signaling in experimental models [34,79]. Conversely, dynamic regulation by m^6^A demethylases and reader proteins fine-tunes the abundance of these transcripts, rendering pyroptosis highly responsive to redox fluctuations.

Mitochondrial dysfunction–induced mtROS accumulation further promotes NLRP3 inflammasome assembly, establishing a redox–inflammation positive feedback loop that amplifies pyroptotic signaling [80]. Within this context, m^6^A functions as a post-transcriptional signal converter, translating redox stress into executable inflammatory death programs.

#### 4.1.2 Amplification of pyroptosis via the m^6^A–mtROS–NLRP3 axis

Pyroptosis proceeds as a cascade rather than a linear event, sustained by multiple amplification mechanisms. In cellular and animal inflammatory models, dysregulation of m^6^A signaling promotes pyroptosis by enhancing mtROS accumulation, suppressing mitophagic clearance of damaged mitochondria, and strengthening inflammatory signal transduction [81]. Impaired MQC allows dysfunctional mitochondria to persistently release mtROS and mitochondrial DNA, which further potentiate NLRP3 inflammasome activation [82,83].

In addition, as discussed in Section 3, m^6^A-regulated non-coding RNAs—including specific lncRNAs and circRNAs—act as secondary amplifiers by modulating NF-κB activity and inflammatory gene transcription. Through these multilayered mechanisms, mtROS generation and pyroptotic susceptibility become self-sustaining under prolonged oxidative stress, explaining the persistence of inflammatory cell death during sepsis.

#### 4.1.3 m^6^A regulation during the execution phase of pyroptosis

During the execution phase of pyroptosis, inflammatory caspase–mediated cleavage of gasdermin D (GSDMD) and subsequent membrane pore formation irreversibly commit cells to lytic death. Although this stage is less directly dependent on mitochondrial signaling, m^6^A modification continues to influence the magnitude and duration of inflammatory cell lysis by regulating the expression of downstream effectors such as GSDMD, IL-1β, and interleukin-18 (IL-18) in cellular and animal inflammatory models [84]. Accordingly, in SIMD, pyroptosis should be viewed not as an isolated immune response but as a mitochondrial redox–initiated and m^6^A-amplified inflammatory death program.

Collectively, m^6^A-mediated post-transcriptional regulation lowers the activation threshold of pyroptosis and amplifies NLRP3–GSDMD–dependent inflammatory cascades under sustained mtROS accumulation. Within the m^6^A–ROS–mitochondrial regulatory axis, pyroptosis represents a predominantly inflammation-amplifying death pathway, which, together with lipid peroxidation–driven ferroptosis, is thought to contribute to irreversible cardiomyocyte injury in sepsis-induced myocardial dysfunction.

### 4.2 m^6^A-regulated ferroptosis: an execution pathway driven by mitochondrial redox imbalance

Ferroptosis is a distinct form of regulated cell death characterized by iron-dependent accumulation of lipid peroxides and is increasingly recognized as a critical downstream consequence of mitochondrial redox imbalance in SIMD [85]. Unlike apoptosis or pyroptosis, ferroptosis more directly reflects the tension between lipid oxidative burden and antioxidant defense capacity, and is tightly linked to mitochondrial metabolic remodeling and excessive ROS generation.

#### 4.2.1 m^6^A amplifies lipid peroxidation stress by regulating core ferroptosis determinants

Accumulating evidence indicates that m^6^A methyltransferases and demethylases modulate ferroptosis susceptibility by controlling the post-transcriptional stability and translation of key ferroptosis-related genes. METTL3-mediated m^6^A modification has been shown to influence the expression of central anti-ferroptotic molecules, including SLC7A11 and glutathione peroxidase 4 (GPX4), thereby weakening GSH-dependent antioxidant defenses and promoting lipid ROS accumulation [73,78,86]. Correspondingly, m^6^A reader proteins, particularly members of the YTHDF family, selectively recognize m^6^A-modified transcripts and accelerate their degradation or translational repression, further amplifying ferroptotic signaling [73].

Beyond METTL3, additional m^6^A regulators have also been implicated in shaping cellular ferroptosis sensitivity [86,87], suggesting that m^6^A does not act on a single target but instead orchestrates a multilayered post-transcriptional network that systematically remodels iron handling, lipid metabolism, and redox balance.

#### 4.2.2 Redox coupling between ferroptosis and mitochondrial dysfunction

Although ferroptosis is not classically defined as a mitochondria-dependent death program, mitochondria function as a potent amplifier of ferroptotic stress under sepsis-associated conditions. m^6^A dysregulation promotes mtROS generation, perturbs iron homeostasis, and disrupts lipid metabolic pathways, thereby indirectly accelerating membrane lipid peroxidation [88]. Impairment of the mETC [89], insufficient NADPH supply [90], and defective fatty acid β-oxidation [91] collectively establish a pro-ferroptotic metabolic environment within the m^6^A-regulated framework.

Accordingly, in SIMD, ferroptosis should be viewed not as a parallel or isolated pathway, but rather as an executional death program that emerges downstream of mitochondrial redox collapse.

#### 4.2.3 Positioning ferroptosis within the m^6^A–ROS–cell fate axis

At the systems level, ferroptosis occupies a downstream position within the m^6^A–ROS–MQC axis, acting alongside pyroptosis and apoptosis to shape the spectrum of cardiomyocyte death. By regulating antioxidant capacity, iron metabolism, and lipid homeostasis, m^6^A influences the relative susceptibility to different death modalities under conditions of sustained oxidative stress [92]. As oxidative pressure escalates, ferroptosis may become a dominant mechanism of irreversible cardiomyocyte injury, accelerating myocardial decompensation.

At a systems level, these findings indicate that m^6^A epitranscriptomic regulation does not merely participate in individual cell death pathways but instead serves as a putative integrative regulatory node that integrates oxidative stress, mitochondrial functional status, and inflammatory signaling to bias cell fate outcomes. In SIMD, sustained oxidative stress initially disrupts mitochondrial homeostasis and promotes mtROS accumulation [93]. In response, m^6^A remodeling alters the post-transcriptional fate of inflammasome components, antioxidant enzymes, and lipid metabolic regulators, thereby redefining cellular sensitivity thresholds to distinct death programs.

Within this unified model, when m^6^A remodeling preferentially enhances inflammatory signaling, inflammasome activation, and GSDMD cleavage, cells are more likely to engage the mtROS–NLRP3–CASP1–GSDMD axis and undergo pyroptosis [62]. In contrast, when m^6^A imbalance predominantly weakens antioxidant defenses and impairs iron and lipid metabolic control, leading to irreversible lipid peroxide accumulation, cells cross a reparative threshold and commit to ferroptosis, defined by iron-dependent lipid ROS overload.

Thus, by dynamically “interpreting” oxidative stress signals at the post-transcriptional level, m^6^A may contribute to a context-dependent bifurcation tendency between pyroptosis and ferroptosis, shaping the heterogeneous landscape of cardiomyocyte loss in sepsis-induced myocardial dysfunction.

### 4.3 m^6^A regulation of apoptosis and necroptosis as secondary cell death pathways

Although the regulatory roles of m^6^A in pyroptosis and ferroptosis have been increasingly characterized, emerging evidence indicates that m^6^A modification also participates in apoptosis and necroptosis, two classical programmed cell death pathways implicated in septic myocardial injury.

In apoptosis, m^6^A modification has been reported to modulate the stability and translation efficiency of transcripts encoding key apoptotic regulators, including BCL-2 family proteins, caspases, and p53 signaling components [[94], [95], [96]]. METTL3-mediated m^6^A deposition can either promote or suppress apoptosis depending on cellular context by altering mRNA decay or translation dynamics. Conversely, demethylases such as FTO and ALKBH5 may fine-tune apoptotic sensitivity by reshaping the epitranscriptomic landscape under oxidative stress conditions [97,98].

Necroptosis, a regulated form of lytic cell death mediated by RIPK1, RIPK3, and MLKL, has also been linked to epitranscriptomic regulation. Recent studies suggest that m^6^A modification can influence inflammatory necrosis by modulating the translation or stability of necroptotic signaling molecules [99]. In septic conditions characterized by excessive TNF-α signaling and redox imbalance, dysregulated m^6^A machinery may alter necroptotic threshold sensitivity, thereby contributing to myocardial inflammatory injury [70]. Although direct evidence in septic myocardium remains limited, these findings support a broader role of m^6^A in orchestrating multiple forms of programmed cell death beyond pyroptosis and ferroptosis.

Thus, m^6^A appears to function as a context-dependent regulator coordinating apoptosis, necroptosis, pyroptosis, and ferroptosis within the redox-stressed myocardial microenvironment. This integrated perspective may help explain the dynamic shift between reversible cardiomyocyte injury and irreversible cell death during SIMD progression.

## 5 Disruption of mitochondrial quality control under m^6^A–ROS regulation

Mitochondria serve as the central hub for energy metabolism and redox homeostasis in cardiomyocytes. Their functional integrity is maintained by the MQC system, which encompasses mitochondrial fusion–fission dynamics, mitophagy, and mitochondrial biogenesis. In SIMD, PAMPs and inflammatory mediators such as TNF-α and IL-1β provoke excessive oxidative stress, leading to collapse of this finely tuned system. This manifests as loss of mitochondrial membrane potential (ΔΨm), excessive ROS generation, impaired ATP synthesis, and progressive mitochondrial structural disintegration.

**Fig. 3:**
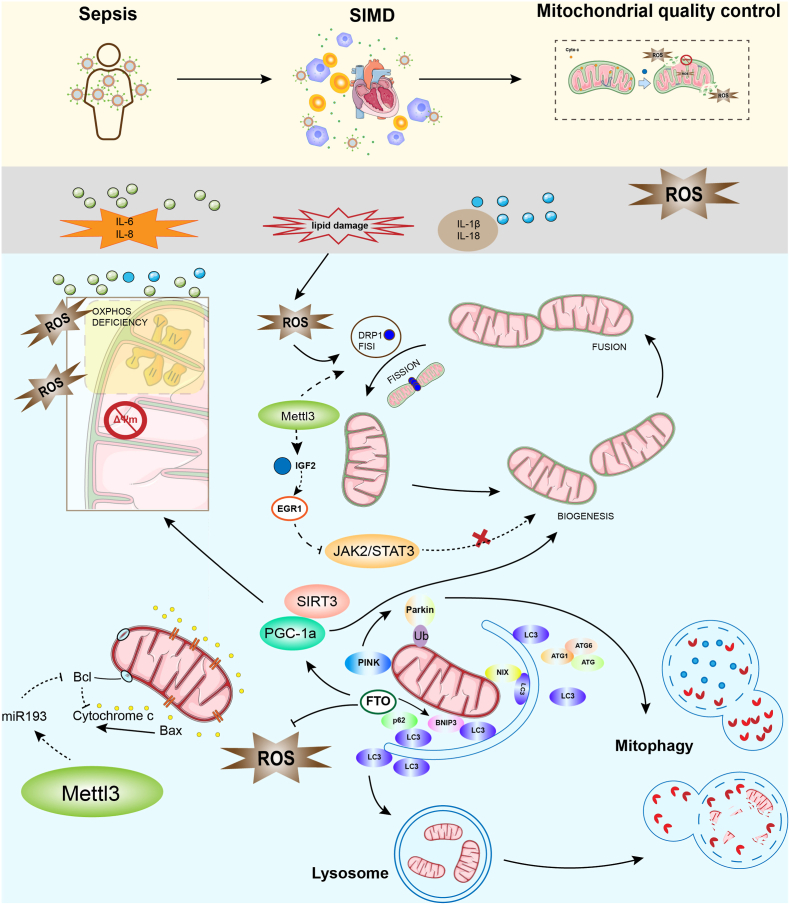
m^6^A-dependent remodeling of mitochondrial quality control links oxidative stress to myocardial injury in sepsis Sepsis-induced systemic inflammation leads to excessive cytokine release and oxidative stress, contributing to SIMD. Persistent inflammatory mediators and ROS converge on cardiomyocytes, triggering mitochondrial dysfunction characterized by impaired OXPHOS, loss of (ΔΨm, and lipid peroxidation. At the epitranscriptomic level, oxidative stress is associated with dysregulation of m^6^A signaling, involving altered activity of m^6^A writers and erasers. METTL3-associated m^6^A remodeling has been implicated in promoting mitochondrial fission through DRP1/FIS1-related pathways and may contribute to suppression of mitochondrial biogenesis via the IGF2–EGR1–JAK2/STAT3 axis, thereby amplifying mitochondrial fragmentation and bioenergetic stress. In contrast, the m^6^A demethylase FTO is associated with preservation of mitochondrial quality control by stabilizing transcripts involved in mitochondrial biogenesis and redox regulation, including PGC-1α and SIRT3. Activation of the SIRT3–PGC-1α pathway supports mitochondrial biogenesis and redox homeostasis, whereas disruption of this axis compromises mitochondrial renewal under septic stress. In parallel, m^6^A-dependent regulation of mitophagy pathways, including PINK1/Parkin- and BNIP3/NIX-mediated mechanisms, governs the clearance of damaged mitochondria through autophagosome–lysosome fusion. Under sustained oxidative stress and m^6^A imbalance, mitophagic capacity may become insufficient, leading to accumulation of dysfunctional mitochondria and persistent ROS generation.

Recent evidence indicates that m^6^A RNA methylation acts as a critical epitranscriptomic regulator of MQC by fine-tuning the post-transcriptional fate of key components, including PTEN-induced kinase 1 (PINK1), FUN14 domain containing 1 (FUNDC1), dynamin-relatedprotein1 (DRP1), mitofusin 2 (MFN2), optic atrophy 1 (OPA1), and PGC-1α [[100], [101], [102], [103], [104], [105]]. Through this mechanism, m^6^A establishes a dynamic signaling network that links oxidative stress, metabolic remodeling, and programmed cell death, ultimately forming a self-reinforcing m^6^A–ROS–MQC–cell death loop in SIMD (Fig. 3).

### 5.1 m^6^A regulation of mitophagy

Mitophagy constitutes a primary defensive barrier that eliminates damaged mitochondria and preserves metabolic homeostasis in cardiomyocytes. Its failure leads to persistent ROS accumulation and mitochondrial DNA (mtDNA) release, thereby triggering inflammatory signaling and pyroptotic responses. During sepsis, ROS accumulation and mitochondrial depolarization induced by PAMPs and systemic inflammation initially activate mitophagic defenses; however, insufficient or dysregulated clearance of damaged mitochondria amplifies oxidative stress and exacerbates myocardial injury [106].

Mitophagy is mainly governed by two signaling paradigms: the ubiquitin-dependent PINK1/Parkin pathway [107], and receptor-mediated mechanisms involving BNIP3, NIP3-like protein X (NIX), and FUNDC1, with the FUNDC1 pathway playing a particularly prominent role under hypoxic and inflammatory stress [108]. FUNDC1-dependent mitophagy has been shown to restrain ROS propagation, attenuate apoptosis, and limit tissue damage in cellular and animal inflammatory or ischemic models [109].

At the epitranscriptomic level, m^6^A modification emerges as a central regulator of mitophagic activity. The m^6^A demethylase FTO exerts a pronounced cardioprotective effect in SIMD. In cellular and animal sepsis or cardiac injury models, downregulation of FTO aggravates mtROS production, impairs respiratory capacity, and destabilizes membrane potential, whereas FTO overexpression restores mitochondrial homeostasis by targeting BNIP3 and activating mitophagic pathways [110]. In contrast, in cardiomyocyte cellular models, aberrant upregulation of the m^6^A writer METTL3 enhances m^6^A modification of pri-miR-193a, promotes its maturation, and suppresses BCL2L2 expression, thereby weakening anti-apoptotic capacity and indirectly compromising mitochondrial stability [75].

Beyond protein-coding transcripts, m^6^A-dependent regulation of non-coding RNAs further shapes mitophagy and mitochondrial homeostasis. In cellular and animal inflammatory or cardiovascular disease models, downregulation of the lncRNA MALAT1 enhances mitophagic flux, stabilizes mitochondrial membrane potential, and reduces ROS generation [42]. In addition, in cellular models, the lncRNA Mir22hg, through YTHDC1-dependent m^6^A recognition, stabilizes Angptl4 transcripts, thereby influencing iron metabolism and mitochondrial homeostasis [78].

Collectively, these findings demonstrate that m^6^A modification regulates mitophagy not merely by modulating autophagic activity per se, but by integrating m^6^A–lncRNA–miRNA–mitophagy regulatory axes. This epitranscriptomic layer constitutes a crucial antioxidant defense mechanism in sepsis and plays a decisive role in maintaining mitochondrial integrity under inflammatory stress.

### 5.2 m^6^A-mediated dysregulation of mitochondrial dynamics

Mitochondrial dynamics are governed by the coordinated actions of fusion proteins (MFN1/2 and OPA1) and the fission machinery centered on DRP1. Disruption of this balance represents a critical node at which oxidative stress is amplified and mitochondrial dysfunction is propagated. In cellular and animal sepsis models, LPS exposure induces mitochondrial membrane depolarization and ROS accumulation in cardiomyocytes, accompanied by excessive activation of the DRP1/Fis1 axis, leading to mitochondrial fragmentation and functional collapse [111]. In cardiomyocyte cellular models, pharmacological inhibition of DRP1, such as with mdivi-1, markedly attenuates ROS production and reduces cardiomyocyte death, underscoring the pathological relevance of fission-dominant remodeling [112].

m^6^A modification plays a pivotal role in reshaping mitochondrial dynamics under stress conditions. In cardiomyocyte cellular and animal hypoxia–ischemia models, suppression of METTL3 reduces the translational efficiency of DRP1 mRNA, resulting in decreased DRP1 protein abundance and mitochondrial fragmentation following hypoxia–ischemia injury, thereby significantly limiting cardiomyocyte death [102]. In addition, in cardiomyocyte cellular models, Wilms tumor 1-associating protein (WTAP)-mediated m^6^A modification of the lncRNA small nucleolar RNA host gene 1 (SNHG1) regulates mitochondrial polarization, mtROS generation, and cardiomyocyte apoptosis through the miR-361-5p/OPA1 axis, ultimately influencing the progression of myocardial ischemia–reperfusion injury [58].

Under hypoxia/reoxygenation (H/R) conditions, METTL3 has also been shown to enhance early growth response 1 (EGR1) expression via insulin like growth factor 2 mRNA binding protein 2 (IGF2BP2) dependent m^6^A recognition, suppressing JAK2/signal transducer and activator of transcription 3 (STAT3) signaling and disrupting mitochondrial dynamic balance. This shift toward excessive fission promotes cardiomyocyte pyroptosis and exacerbates ischemia–reperfusion injury in cardiomyocyte cellular and animal ischemia–reperfusion models [113]. Consistently, in endotoxemia cellular and animal models, during endotoxemia, coordinated downregulation of MFN2 and upregulation of DRP1 has been observed, resulting in sustained mitochondrial fragmentation and progressive ROS accumulation [112]. Furthermore, in cellular and animal inflammatory or cardiac injury models, CaMKIV-mediated phosphorylation of DRP1 at Ser616 suppresses MFN1/2 and OPA1 expression, reinforcing a fission-dominant state and amplifying oxidative stress [114].

Collectively, these findings indicate that m^6^A-dependent post-transcriptional regulation acts as a molecular switch that biases mitochondrial dynamics toward pathological fission under septic stress, thereby accelerating ROS amplification and mitochondrial failure.

### 5.3 m^6^A regulation of mitochondrial energy metabolism and oxidative stress amplification

Mitochondrial bioenergetic failure represents a central pathological feature of SIMD. During sepsis, impairment of OXPHOS leads to ATP depletion and excessive ROS generation, which in turn trigger multiple forms of programmed cell death [115]. Genetic deletion of DNA-dependent protein kinase (DNA-PK) alleviates myocardial injury by restoring metabolic balance and reducing ROS burden, highlighting the tight coupling between energy metabolism and redox homeostasis in animal models of sepsis or endotoxemia [116]. Similarly, the transforming growth factor-β superfamily member growth differentiation factor 15 (GDF15) enhances antioxidant defenses and suppresses ferroptosis through the ALK5–SMAD2/3–JAK2/STAT3–GPX4 signaling axis, thereby protecting against septic cardiac injury in cellular and animal models of sepsis [117].

The translocator protein (TSPO) has emerged as an important modulator of mitochondrial redox balance in sepsis. TSPO is markedly upregulated in cardiomyocytes during septic stress, where it disrupts membrane integrity, promotes excessive ROS production, activates RIP1/RIP3 signaling, impairs autophagic flux, and amplifies inflammatory injury in cellular models of septic cardiomyocyte injury [118]. Notably, NADH, a TSPO-binding molecule, restores autophagic flux and mitochondrial function, identifying the TSPO–ROS–autophagy axis as a potentially druggable therapeutic circuit in SIMD [118].

At the metabolic signaling level, LPS suppresses the peroxisome proliferator-activated receptor-α (PPAR–PGC-1α) pathway, impairing fatty acid transport into mitochondria and promoting lipid accumulation and lipotoxicity [[119], [120], [121], [122]]. In septic mouse hearts, global m^6^A RNA methylation is increased, concomitant with reduced expression of the demethylase FTO, an enzyme closely linked to metabolic regulation [72]. These epitranscriptomic alterations correlate with marked upregulation of inflammatory cytokine genes (including IL-6, TNF-α, and IL-1β) and deterioration of left ventricular function in this animal model [72]. Consistently, in LPS-treated H9C2 cardiomyocyte cellular models, a similar pattern is observed, characterized by elevated m^6^A levels, enhanced inflammatory gene expression, and downregulation of FTO [72].

Together, these data suggest that m^6^A remodeling couples metabolic reprogramming to redox imbalance in SIMD. By influencing mitochondrial substrate utilization, antioxidant capacity, and inflammatory signaling, m^6^A dysregulation drives a vicious cycle in which energy failure and oxidative stress mutually reinforce each other, ultimately converging on irreversible cardiomyocyte death.

### 5.4 m^6^A regulation of mitochondrial biogenesis

Mitochondrial biogenesis refers to the coordinated synthesis of new mitochondria and is essential for maintaining cardiomyocyte function under high metabolic demand and during stress adaptation. This process is tightly controlled by a transcriptional network centered on peroxisome proliferator-activated receptor gamma coactivator 1α (PGC-1α), nuclear respiratory factors 1 and 2 (NRF1/2), and mitochondrial transcription factor A (TFAM). As the master regulator, PGC-1α cooperates with NRF1/2 to induce TFAM transcription, thereby promoting mitochondrial DNA replication, mitochondrial protein synthesis, and the restoration of OXPHOS capacity.

Accumulating clinical and experimental evidence suggests that early activation of mitochondrial biogenesis is critical for survival in sepsis-induced critical illness. In clinical studies of septic patients, survivors of sepsis exhibit higher skeletal muscle ATP levels, a reduced phosphocreatine-to-ATP ratio, and early induction of both mitochondrial biogenesis and antioxidant defense programs, underscoring the adaptive value of this response under systemic inflammatory stress [123].

Inflammatory and metabolic signaling pathways critically modulate mitochondrial biogenesis. Cyclooxygenase-2 (COX-2) inhibition suppresses PGC-1α expression and attenuates mitochondrial biogenesis, whereas Prostaglandin E2 (PGE2) enhances mitochondrial biogenesis through PGC-1α activation in experimental cellular and animal models [124]. In LPS-treated cardiomyocyte cellular models, reduced expression of sirtuin 3 (SIRT3) and AMP-activated protein kinase (AMPK) precedes mitochondrial dysfunction and cell death [125]. Conversely, in cellular and animal models, SIRT3 overexpression restores AMPK signaling, promotes mitochondrial biogenesis, preserves redox homeostasis, and suppresses mitochondria-dependent apoptosis. Importantly, pharmacological or genetic inhibition of mitochondrial biogenesis in these models abolishes the cardioprotective effects of the SIRT3–AMPK axis, highlighting mitochondrial renewal as a requisite component of stress resistance [125].

In myocardial injury models, PGC-1α has been identified as a downstream target of the m^6^A demethylase FTO. In cardiomyocyte cellular and animal myocardial injury models, by removing m^6^A marks, FTO enhances PGC-1α mRNA stability and sustains mitochondrial biogenesis [76]. In contrast, in cardiomyocyte cellular models, METTL3-driven m^6^A modification promotes the biogenesis of miR-503, which directly targets PGC-1β and SIRT3, thereby impairing mitochondrial metabolism and accelerating cardiomyocyte death [126]. These findings illustrate that m^6^A-dependent RNA regulation can exert bidirectional effects on mitochondrial biogenesis depending on the balance between protective and deleterious downstream pathways.

Under septic conditions, persistent exposure to inflammatory cytokines such as TNF-α and IL-6 suppresses PGC-1α transcriptional activity and disrupts the m^6^A regulatory network, leading to reduced stability and translational efficiency of mitochondrial biogenesis-related mRNAs [127]. Consequently, the renewal and functional recovery of damaged mitochondria are severely compromised. In sepsis models, ginsenoside Rg1 has been shown to suppress F-box protein 3 (FBXO3) expression and reduce PGC-1α ubiquitination in an m^6^A–YTHDF1-dependent manner [128]. In LPS-treated cellular models, overexpression of FBXO3 abolishes the protective effects of Rg1 on LPS-induced epithelial inflammation, apoptosis, and mitochondrial dysfunction, highlighting the interplay between m^6^A modification, ubiquitin–proteasome signaling, and mitochondrial biogenesis in inflammatory injury [128].

Collectively, MQC constitutes a central defense system that enables cells to adapt to oxidative stress and inflammatory insults by coordinating mitophagy, mitochondrial dynamics, metabolic remodeling, and biogenesis. In sepsis-induced myocardial dysfunction, sustained inflammation and oxidative stress disrupt m^6^A-dependent post-transcriptional regulation, leading to a loss of coordination among MQC modules. As damaged mitochondria accumulate and clearance efficiency declines, excessive mtROS production and metabolic imbalance exceed the cellular compensatory threshold, driving irreversible cell fate decisions.

In this context, MQC failure does not represent the terminal event but rather serves as the bifurcation point for programmed cell death. When m^6^A remodeling preferentially amplifies inflammatory sensing and inflammasome signaling, accumulated mtROS promotes pyroptosis via the NLRP3–CASP1–GSDMD axis. Conversely, when m^6^A dysregulation predominantly compromises antioxidant defenses, iron homeostasis, and lipid metabolism, mitochondrial lipid peroxidation pressure drives cells toward ferroptotic death. Thus, m^6^A-mediated MQC disruption functions as a critical integrative hub linking mitochondrial dysfunction to multiple programmed cell death pathways, providing a unified mechanistic framework for understanding the heterogeneous cell fate outcomes observed in sepsis-induced myocardial dysfunction (see Table 2).

**Table 2:** m^6^A-mediated regulation of mitochondrial quality control (MQC) in sepsis-induced myocardial dysfunction.

MQC module	Key molecular events	m^6^A regulators involved	Downstream mitochondrial consequences	Functional outcomes in SIMD	Ref.
**Mitophagy** (PINK1/Parkin; FUNDC1-dependent)	Impaired clearance of damaged mitochondria under excessive ROS	FTO; METTL3; YTHDC1; YTHDF1	Accumulation of dysfunctional mitochondria; mtROS amplification; mtDNA release	Enhanced oxidative stress, inflammasome activation, cardiomyocyte death	[42,75,78,[107], [108], [109], [110]]
	FTO-mediated demethylation stabilizes BNIP3 mRNA	FTO	Restoration of mitophagy flux; reduced mtROS	Cardioprotection, improved mitochondrial homeostasis	[110]
	m^6^A-modified lncRNAs (e.g., MALAT1, Mir22hg) regulate mitophagy	METTL3; YTHDC1	Altered autophagic clearance and iron metabolism	Modulation of oxidative injury and cell survival	[42,78]
**Mitochondrial dynamics** (Fusion–fission balance)	Excessive DRP1 activation and mitochondrial fragmentation	METTL3; WTAP; IGF2BP2	Network fragmentation; ΔΨm collapse; ROS burst	Exacerbation of myocardial injury and cell death	[58,111,113]
	m^6^A-dependent translation of Drp1 mRNA	METTL3	Enhanced fission, impaired bioenergetics	Increased susceptibility to ischemic or septic injury	[102]
	lncRNA–miRNA axis regulating OPA1/MFN2	WTAP; m^6^A-modified lncRNAs	Suppressed fusion capacity	ROS accumulation, apoptosis/pyroptosis	[58,114]
**Mitochondrial energy metabolism**	OXPHOS inhibition and ATP depletion	FTO; global m^6^A	Reduced NADPH and antioxidant capacity	Metabolic failure, ROS amplification	[72,116]
	TSPO overexpression disrupts membrane integrity	Indirect m^6^A involvement	Impaired autophagic flux; excessive ROS	Inflammation and necroptosis activation	[118]
	PPAR–PGC-1α signaling suppression	m^6^A-dependent destabilization	Lipid accumulation and lipotoxicity	Contractile dysfunction	[[119], [120], [121], [122]]
**Mitochondrial biogenesis**	Suppressed PGC-1α/NRF1/TFAM axis under inflammation	METTL3; FTO; YTHDF1	Impaired mitochondrial renewal and recovery	Persistent mitochondrial dysfunction	[76,[123], [124], [125]]
	m^6^A-dependent translation of PGC-1α and TFAM	METTL3; YTHDF1	Context-dependent enhancement of biogenesis	Adaptive or maladaptive remodeling	[76]
	miR-503 biogenesis driven by METTL3	METTL3	Inhibition of PGC-1β and SIRT3	Metabolic collapse and cardiomyocyte death	[126]
	FBXO3–m^6^A–YTHDF1 axis modulates PGC-1α ubiquitination	YTHDF1	Stabilization of PGC-1α protein	Improved mitochondrial function and survival	[128]

## 6 Therapeutic strategies targeting the m^6^A–mitochondrial axis

The evidence reviewed above indicates that SIMD is driven by sustained oxidative stress and progressive mitochondrial collapse, representing an energy-crisis–type cell death program orchestrated by dysregulated m^6^A epitranscriptomic networks. Restoring the dynamic balance of the m^6^A–ROS–MQC axis therefore emerges as a promising translational strategy to interrupt the vicious “epigenetic–metabolic–cell death” cycle in SIMD.

### 6.1 Targeting the m^6^A machinery

The reversibility of m^6^A modification makes it an attractive therapeutic target. In cellular and animal disease models, pharmacological inhibition of the m^6^A “writer” METTL3 (e.g., STM2457, UZH1a) has shown anti-inflammatory and mitochondria-protective effects in multiple disease models [129,130]. Notably, in animal models of sepsis-associated lung injury, STM2457 attenuates the m^6^A modification of NLRP3, acyl-CoA synthetase long chain family member 4 (ACSL4), and caspase 9 (CASP9) transcripts, thereby potentially dampening the amplification of pyroptotic and ferroptotic signaling, suppressing ferroptosis, and alleviating sepsis-associated lung injury [131]. Conversely, in cellular and animal models, activation of the m^6^A “eraser” FTO (e.g., FB23-2, CS1) has been reported to restore the stability of antioxidant- and autophagy-related mRNAs such as SLC7A11, PGC-1α, and FUNDC1, reducing ROS accumulation and mitochondrial fragmentation [77].

Interventions targeting m^6^A “reader” proteins have also gained attention. Limiting YTHDF1 binding to pro-inflammatory or pro-apoptotic transcripts may achieve a post-transcriptional “de-amplification” effect, restraining excessive inflammatory and cell-death signaling [132,133]. In contrast, YTHDF2 functions as a critical mRNA “scavenger”: by accelerating the decay of transcripts encoding NLRP3, IL-6, and caspase-3, it effectively prevents pathological signal overactivation [134]. Consistently, YTHDF2 deficiency is associated with exacerbated inflammation or apoptosis [[135], [136], [137]], highlighting YTHDF2 enhancement as a potential protective strategy. YTHDF3, acting cooperatively with YTHDF1/2 under specific stress conditions [138], further modulates the balance between ferroptosis and autophagy, contributing to tissue homeostasis [139]. It should be noted that most current pharmacological evidence targeting m^6^A regulators is derived from non-cardiac or non-SIMD models, and their direct efficacy and safety in septic myocardium remain to be established.

### 6.2 Restoring mitochondrial quality control

Given the deep integration of m^6^A signaling into MQC networks, re-establishing MQC homeostasis represents a key step in disrupting the pathological feedback loop. m^6^A modification frequently enhances DRP1 translation or stability, thereby promoting mitochondrial fission. When unchecked, excessive fission disrupts mitochondrial network integrity, precipitating energetic failure and cell death. In contrast, promoting mitochondrial fusion (via MFN2/OPA1) and restraining excessive fission (via DRP1 inhibition) can reverse the structural damage induced by the m^6^A–DRP1 axis [102,140].

Concurrently, activation of PINK1- or FUNDC1-mediated mitophagy—achieved by agents such as melatonin, resveratrol, or nicotinamide mononucleotide (NMN)—significantly reduces damaged mitochondria and ROS burden, while restoring mitochondrial membrane potential (ΔΨm) and ATP production [[141], [142], [143], [144]]. These approaches effectively interrupt the positive feedback loop linking m^6^A dysregulation, ROS accumulation, MQC failure, and cell death, thereby re-establishing mitochondrial energetic and redox homeostasis.

### 6.3 Multi-target advantages of western and traditional Chinese medicine interventions

Both Western pharmacotherapy and traditional Chinese medicine (TCM)–derived compounds have been explored for their potential to mitigate mitochondrial dysfunction and oxidative stress during sepsis. Jiang and colleagues developed a human serum albumin (HSA)–based self-assembled nanocomposite combining cerium nanozymes and curcumin (CeCH), which suppresses sepsis-induced cardiac injury by inhibiting ferroptosis and inflammation in preclinical cellular and animal models [145]. Importantly, the cardioprotective effects of CeCH have been primarily attributed to its ROS-scavenging capacity and modulation of inflammatory responses, rather than direct regulation of the m^6^A machinery. CeCH nanoparticles promote M2 macrophage polarization, scavenge ROS, reduce inflammatory responses in vitro, and, in LPS-induced sepsis models, markedly attenuate ferroptosis, suppress pro-inflammatory cytokine release, and restore cardiac function [145].

Statins have also been reported to modulate inflammation, endothelial function, and mitochondrial metabolism during sepsis, potentially through PPAR-α–dependent or independent mechanisms in experimental models [146]. However, current evidence does not directly link statin-mediated cardioprotection to m^6^A-dependent epitranscriptomic regulation, and their effects are more plausibly explained by broad anti-inflammatory and metabolic actions.

Similarly, several natural compounds commonly used in TCM, including quercetin, puerarin, and resveratrol, have been shown to attenuate oxidative stress and preserve mitochondrial integrity in experimental sepsis or inflammatory models. In some non-cardiac or non-septic systems, these agents have been reported to influence components of the m^6^A regulatory network, such as the NRF2–FTO or METTL3-related pathways [[147], [148], [149]]. However, direct evidence demonstrating that these compounds modulate myocardial m^6^A machinery in sepsis remains limited. Thus, their reported cardioprotective effects in SIMD are more likely mediated through upstream redox modulation and mitochondrial protection, rather than direct targeting of m^6^A writers, erasers, or readers.

While targeting the m^6^A–mitochondrial axis represents a promising therapeutic strategy for SIMD, it is important to recognize that m^6^A regulators such as METTL3 and FTO are ubiquitously expressed and participate in essential physiological processes in normal cardiomyocytes. Basal m^6^A modification contributes to mitochondrial biogenesis, metabolic flexibility, and stress adaptation under physiological conditions. Therefore, indiscriminate inhibition of METTL3 or systemic activation of FTO may disrupt mitochondrial homeostasis, impair ATP production, or alter redox balance in non-diseased myocardium. Moreover, given that m^6^A modification regulates a broad spectrum of transcripts beyond mitochondrial pathways, global modulation of m^6^A enzymes may produce unintended cardiovascular consequences, including maladaptive hypertrophic signaling, altered calcium handling, or vascular dysfunction. These potential off-target effects underscore the need for precision-based intervention strategies. To minimize adverse effects, future therapeutic development should prioritize context-dependent modulation rather than complete enzymatic blockade. Strategies such as cardiomyocyte-specific delivery systems, inducible or phase-specific modulation during the hyperinflammatory or sustained injury stages of sepsis, and targeting downstream effector transcripts within the m^6^A–mitochondrial network may offer greater safety profiles. In addition, small molecules that fine-tune m^6^A activity within a physiological range, rather than fully suppressing or activating core enzymes, may preserve basal mitochondrial function while correcting pathological reprogramming.

From a translational perspective, effective targeting of the m^6^A–mitochondrial axis in SIMD will require careful consideration of timing, cell-type specificity, and delivery strategy. Early-phase interventions may aim to preserve mitochondrial quality control and antioxidant capacity, whereas late-stage modulation may need to restrain irreversible inflammatory or ferroptotic signaling. Biomarker-guided patient stratification based on redox status, mitochondrial dysfunction, or circulating m^6^A signatures may further enable precision intervention. Ultimately, context-dependent and tissue-restricted modulation of m^6^A pathways, rather than global enzymatic inhibition or activation, is likely to offer the most favorable therapeutic window.

## 7 Current limitations and challenges

Although accumulating evidence suggests that m^6^A modification may play an important regulatory role in SIMD, research in this field remains at an early stage and is confronted with several conceptual and technical challenges. These limitations can be summarized as follows.

### 7.1 Limited overall evidence and lack of systematic investigation

Current studies linking m^6^A modification to SIMD are largely fragmented and predominantly associative. Most reports focus on descriptive changes in the expression of m^6^A-related enzymes, such as METTL3 or FTO, without systematically defining their downstream targets, signaling pathways, or functional consequences. A coherent causal framework linking m^6^A remodeling with downstream target transcripts and myocardial injury phenotypes has not yet been firmly established. Future studies should focus on establishing causal regulatory chains linking specific m^6^A-modified transcripts to mitochondrial dysfunction, inflammatory signaling, and myocardial injury phenotypes using loss- and gain-of-function models combined with transcript-specific m^6^A editing strategies.

### 7.2 Challenges in m^6^A target identification and limited specificity

High-throughput approaches, including MeRIP-seq and m^6^A-seq, have facilitated the global mapping of m^6^A modifications; however, their spatial resolution and quantitative accuracy remain suboptimal, making it difficult to pinpoint functionally critical modification sites. Moreover, m^6^A marks are highly dynamic and context dependent, with substantial variability across pathological states, further complicating accurate target selection in SIMD. The development of single-nucleotide resolution m^6^A mapping technologies, such as miCLIP, m6A-REF-seq, and direct RNA sequencing, together with site-directed mutagenesis and transcript-specific rescue experiments, will be essential to identify functionally relevant m^6^A sites in mitochondrial and cell death–related transcripts in SIMD.

### 7.3 Mechanistic complexity and unresolved regulatory networks

m^6^A exerts its effects through a multilayered “writer–eraser–reader” system, influencing RNA splicing, nuclear export, stability, and translational efficiency. Crosstalk among different m^6^A enzymes and the context-dependent engagement of distinct reader proteins can lead to divergent biological outcomes. In the setting of sepsis-related myocardial injury, the architecture, hierarchy, and functional specialization of these regulatory networks remain poorly defined. Future research should therefore move beyond single-gene studies toward network-level analyses integrating m^6^A regulation with mitochondrial quality control, redox signaling, and inflammatory pathways, in order to define the hierarchical structure of the m^6^A–mitochondria regulatory network in SIMD.

### 7.4 Limitations of animal and cellular models

Most available data are derived from acute LPS-induced sepsis models or cecal ligation and puncture (CLP) models, which only partially recapitulate the clinical complexity and temporal heterogeneity of human sepsis. In addition, widely used H9c2 cells do not fully represent mature cardiomyocytes, exhibiting distinct metabolic properties and epitranscriptomic profiles compared with adult myocardial cells. These limitations constrain the translational relevance of current findings. The establishment of clinically relevant sepsis models with temporal resolution, as well as validation in human myocardial tissue, circulating biomarkers, or induced pluripotent stem cell–derived cardiomyocytes, will be critical to improve the translational relevance of m^6^A research in SIMD.

### 7.5 Lack of validated m^6^A-targeted therapeutic strategies

Although several pharmacological modulators of the m^6^A pathway—such as experimental METTL3 inhibitors (e.g., STM2457)—have been developed, their efficacy and safety in cardiac diseases, particularly in SIMD, remain largely unexplored. Most interventional studies are confined to in vitro settings, with a clear lack of systematic in vivo validation and translational investigation. Future therapeutic development should focus on improving the specificity and delivery of m^6^A modulators, particularly through cardiac-targeted nanoparticle systems, RNA-guided epitranscriptomic editing, and stage-specific intervention strategies, to enhance therapeutic efficacy while minimizing systemic side effects.

### 7.6 Insufficient understanding of crosstalk with other epigenetic mechanisms

m^6^A modification does not operate in isolation but interacts extensively with other epigenetic and epitranscriptomic processes, including histone modifications, DNA methylation, and non-coding RNA–mediated regulation. How these regulatory layers integrate to shape inflammatory signaling, mitochondrial dysfunction, and cardiomyocyte injury in sepsis remains largely unknown, highlighting a major gap in current research. Integrative multi-omics approaches combining epigenomics, epitranscriptomics, metabolomics, and mitochondrial proteomics will be necessary to elucidate how these regulatory layers interact to control mitochondrial homeostasis and inflammatory injury in sepsis.

### 7.7 Histone lactylation–m^6^A crosstalk as an emerging epigenetic challenge in SIMD

Beyond RNA methylation itself, increasing attention has been directed toward potential crosstalk between m^6^A epitranscriptomic regulation and other metabolite-driven epigenetic mechanisms. Histone lactylation, a recently identified post-translational modification linking glycolytic flux to transcriptional activation, has emerged as a candidate interface connecting metabolic stress to gene regulatory programs [150]. In the context of sepsis, profound metabolic rewiring leads to excessive lactate accumulation in cardiomyocytes and infiltrating immune cells [151], raising the possibility that histone lactylation may participate in shaping the epigenetic landscape of the septic heart [150].

Although direct evidence for histone lactylation–m^6^A coupling in sepsis-induced myocardial dysfunction is currently lacking, several conceptual models support a potential interaction between these two regulatory layers. First, lactylation-mediated chromatin remodeling may alter the transcriptional accessibility of loci encoding core m^6^A regulators, such as METTL3, FTO, and ALKBH5, thereby indirectly modulating the abundance and balance of the m^6^A machinery [25,152]. Second, changes in chromatin openness and RNA polymerase II elongation dynamics induced by histone lactylation could influence co-transcriptional m^6^A deposition on nascent transcripts, particularly those involved in mitochondrial quality control, redox signaling, and regulated cell death pathways [[153], [154], [155], [156]].

Notably, many genes governing mitophagy, inflammasome activation, and stress-responsive cell death undergo rapid and robust transcriptional induction during sepsis. In such a context, lactylation-driven chromatin permissiveness may create a transcriptional environment that favors enhanced m^6^A deposition, thereby fine-tuning RNA fate decisions under conditions of overwhelming metabolic and oxidative stress. This model suggests that histone lactylation could act upstream of m^6^A remodeling, integrating metabolic cues with post-transcriptional control of mitochondrial homeostasis and cardiomyocyte survival.

Future studies should prioritize investigating the interaction between histone lactylation and m^6^A remodeling using integrated chromatin profiling, single-nucleotide resolution m^6^A mapping, and cell type–specific functional perturbation approaches. Emerging technologies such as single-cell epitranscriptomics, time-resolved sepsis models, and integrative multi-omics analyses combining epigenomics, metabolomics, and mitochondrial proteomics will be essential to define whether histone lactylation and m^6^A operate as coordinated layers within a unified metabolic–epigenetic regulatory network in SIMD. Clarifying this regulatory interface may uncover new intervention points for restoring mitochondrial function and limiting myocardial injury in sepsis.

Box 1 **Experimental Evidence Supporting the m6A–ROS–Mitochondrial Axis in Sepsis-Induced Myocardial Dysfunction.**

**Evidence directly demonstrated in SIMD**

● Mitochondrial dysfunction, excessive reactive oxygen species (ROS) accumulation, and impaired myocardial energy metabolism are consistently observed in both experimental models and clinical cases of SIMD.
● Activation of multiple forms of programmed cell death, including pyroptosis, apoptosis, and ferroptosis-like injury, has been directly documented in septic myocardium and cardiomyocytes.
● Altered expression of core m^6^A regulatory enzymes, such as METTL3, METTL14, FTO, and ALKBH5, has been reported in cardiac tissue and isolated cardiomyocytes under septic conditions.
● Disruption of MQC processes, including mitophagy and mitochondrial dynamics, closely correlates with oxidative stress severity and myocardial dysfunction in SIMD.

**Evidence inferred from related sepsis models or non-cardiac systems**

● m^6^A-dependent regulation of inflammasome activation, pyroptosis, and ferroptosis has been mechanistically demonstrated in immune cells, endothelial cells, and non-cardiac tissues exposed to septic or inflammatory stress.
● Epitranscriptomic control of mitochondrial function, ROS generation, and metabolic reprogramming by m^6^A has been extensively characterized in ischemia–reperfusion injury, heart failure, and other cardiovascular or inflammatory diseases.
● Comparative studies in sepsis-associated acute kidney injury and acute lung injury reveal organ-specific m^6^A-regulated inflammatory and oxidative stress pathways, providing an inferential framework for understanding tissue-dependent regulation.
● Direct causal links between specific m^6^A modifications and defined cardiomyocyte death modalities in SIMD remain limited and are largely extrapolated from these related systems.

**Interpretive note**

● The m^6^A–ROS–mitochondrial axis proposed in this review represents a hypothesis-driven integrative framework that synthesizes direct evidence from SIMD with mechanistic insights inferred from related pathological contexts.
● Future studies employing cardiomyocyte-specific, stage-resolved, and single-cell approaches will be required to validate and refine this model in septic myocardial injury.

## 8 Discussion

This review synthesizes emerging evidence and positions the m^6^A–ROS–MQC axis as a hypothesis-driven, systems-level framework for understanding sepsis-induced myocardial dysfunction. Rather than viewing m^6^A as an isolated epitranscriptomic modifier, we conceptualize it as a redox-sensitive integrator that translates oxidative stress signals into coordinated mitochondrial quality control remodeling and context-dependent modulation of programmed cell death.

Importantly, we propose that m^6^A regulation should not be viewed simply as “upregulating” or “downregulating” specific biological processes. Instead, m^6^A is proposed to rewire the system-level architecture of oxidative stress signaling, thereby enabling context-dependent biasing of cell fate trajectories between distinct death modalities, such as pyroptosis and ferroptosis. When m^6^A remodeling preferentially amplifies inflammatory sensing and inflammasome signaling, mitochondrial redox cues are more readily translated into inflammatory death instructions. Conversely, when m^6^A imbalance predominantly weakens antioxidant defenses and lipid homeostasis, cells are biased toward iron-dependent lipid peroxidation and ferroptotic death. This conceptual framework provides a plausible and testable explanatory model for the pronounced heterogeneity of cardiomyocyte fate observed in SIMD.

Despite the increasing conceptual coherence of this framework, the causal role of m^6^A regulation in SIMD must be interpreted with caution. A central unresolved question is whether m^6^A remodeling primarily acts as an upstream driver of mitochondrial dysfunction or represents a downstream adaptive response to oxidative stress. Current evidence suggests that these two roles are not mutually exclusive but are likely to operate in a time- and context-dependent manner during sepsis progression. Most current evidence is derived from in vitro systems or whole-animal models, making it difficult to determine whether m^6^A alterations represent secondary adaptive responses to oxidative stress or act as upstream determinants actively shaping cell fate. Resolving this causality will require experimental strategies with high temporal and cell-type specificity, including conditional cardiac-specific manipulation of core m^6^A regulators and time-resolved intervention studies that selectively perturb m^6^A remodeling before or after the onset of mitochondrial dysfunction. Moreover, reported changes in key m^6^A regulators—such as METTL3, METTL14, FTO, and ALKBH5—are not fully consistent across sepsis models. These discrepancies likely reflect differences in disease stage, cell type, and experimental context rather than experimental bias alone.

Another critical issue is the pronounced spatiotemporal specificity of m^6^A-mediated regulation. During the early phase of acute inflammation, m^6^A-driven post-transcriptional remodeling may support mitochondrial function and buffer oxidative injury. In contrast, during prolonged or decompensated stages, the same regulatory machinery may amplify inflammatory signaling and promote irreversible cell death. The precise time windows, threshold conditions, and molecular switches governing this transition from protection to injury remain largely unexplored, limiting current assessments of m^6^A-targeted interventions. In addition, the relationship between m^6^A regulation and distinct forms of programmed cell death remains incompletely resolved. Although increasing evidence suggests that m^6^A participates in the regulation of pyroptosis, ferroptosis, and apoptosis, it is unclear whether these effects converge through shared upstream redox signaling or operate independently in a context-specific manner. This uncertainty is further compounded by cell-type specificity, as cardiomyocytes, endothelial cells, and immune cells may exhibit fundamentally different responses to m^6^A dysregulation during sepsis, adding another layer of mechanistic complexity.

Importantly, m^6^A operates within a broader epitranscriptomic landscape rather than in isolation. Other RNA modifications, including N^1^-methyladenosine (m^1^A), 5-methylcytosine (m^5^C), pseudouridine, and adenosine-to-inosine RNA editing, have been implicated in the regulation of RNA stability, translation, and stress responsiveness [157,158]. Emerging evidence suggests that these modifications may influence inflammatory signaling, oxidative stress adaptation, and mitochondrial gene expression [158]. Although direct links between these RNA modifications and sepsis-induced myocardial dysfunction remain limited, potential crosstalk among multiple epitranscriptomic layers may collectively shape mitochondrial homeostasis and cell fate decisions under septic stress.

While the present review focuses on septic myocardial injury, accumulating evidence from other sepsis-associated organ injuries provides an important comparative and inferential framework to better define the specificity of m^6^A regulation in SIMD. In sepsis-induced acute kidney injury (AKI), m^6^A dysregulation has been reported to primarily affect tubular epithelial cell apoptosis and inflammatory signaling, often through modulation of NF-κB–related transcripts and oxidative stress pathways [159]. In contrast, studies in sepsis-associated acute lung injury (ALI) suggest that m^6^A remodeling predominantly influences endothelial barrier integrity and macrophage polarization [160,161]. Compared with these organs, the heart exhibits a uniquely high dependence on mitochondrial OXPHOS and tightly coordinated MQC. Accordingly, whether and how these non-cardiac m^6^A regulatory paradigms translate to cardiomyocytes under septic conditions remains an open and testable question.

Therapeutically, the m^6^A–redox–mitochondrial axis offers a novel conceptual framework for SIMD intervention, but clinical translation will require highly cautious and stratified strategies. Unlike conventional broad-spectrum antioxidant or anti-inflammatory therapies, targeting m^6^A regulation may act by reshaping how cells interpret oxidative stress, rather than simply suppressing stress signals themselves. Given the strong cell-type and stage specificity of m^6^A regulation, future interventions must precisely define therapeutic windows, target cell populations, and regulatory directionality to avoid disrupting early protective responses. Viewing m^6^A as a plastic regulatory hub rather than a single druggable target may therefore be essential for advancing translational applications in SIMD.

However, several major barriers currently limit the clinical translation of m^6^A-targeted therapies in sepsis-induced myocardial dysfunction. First, m^6^A regulation exhibits strong cell-type specificity, and systemic modulation of m^6^A regulators may produce heterogeneous or even opposing effects in cardiomyocytes, endothelial cells, and immune cells. Second, m^6^A remodeling is highly stage-dependent during sepsis progression, and inappropriate timing of intervention may disrupt adaptive stress responses rather than provide protection. Third, m^6^A regulators participate in multiple physiological processes beyond mitochondrial regulation, raising concerns regarding off-target effects and systemic toxicity. In addition, reliable clinical biomarkers reflecting m^6^A activity and epitranscriptomic remodeling are currently lacking, which complicates patient stratification and therapeutic monitoring. Moreover, the systemic nature of m^6^A regulation raises additional concerns regarding safety and unintended transcriptional reprogramming, as m^6^A regulators control a large number of transcripts across multiple organs. In critically ill patients with sepsis, where metabolic and immune states are highly unstable, systemic manipulation of m^6^A pathways may produce unpredictable effects. Therefore, future translational strategies should prioritize organ-targeted delivery systems, stage-specific intervention windows, and biomarker-guided patient stratification.

Another important issue that may influence the clinical application of m^6^A-targeted therapies is the heterogeneity of sepsis. Sepsis is not a uniform disease but a syndrome with substantial variability in inflammatory intensity, immune status, metabolic profile, and organ involvement. Emerging evidence suggests that m^6^A regulation is highly context-dependent and may differ across sepsis subtypes, disease stages, and affected organs. For example, in hyperinflammatory sepsis, m^6^A remodeling may preferentially amplify inflammatory signaling and pyroptotic pathways, whereas in immunosuppressive phases, altered m^6^A regulation may affect immune cell survival, metabolic adaptation, and tissue repair processes. Similarly, mitochondrial dysfunction and m^6^A-dependent regulation may differ between cardiac, renal, pulmonary, and vascular tissues, reflecting organ-specific metabolic demands and stress responses. This heterogeneity has important implications for therapeutic strategy selection. Inflammatory-dominant sepsis may benefit from inhibition of m^6^A writers that amplify inflammatory signaling, whereas conditions characterized by mitochondrial failure or metabolic collapse may require restoration of m^6^A-dependent mitochondrial quality control and biogenesis pathways. Importantly, different forms of sepsis may involve distinct dominant pathological processes, including hyperinflammatory sepsis, immunosuppressive sepsis, septic shock characterized by severe metabolic collapse, and organ-dominant sepsis such as septic cardiomyopathy or septic acute kidney injury. Because m^6^A regulation is closely linked to metabolic state, inflammatory signaling, and mitochondrial function, the direction and magnitude of m^6^A remodeling are likely to differ across these sepsis phenotypes. This context dependency may partly explain why m^6^A regulators exhibit protective roles in some studies but detrimental roles in others, and highlights the importance of precision epitranscriptomic strategies tailored to specific sepsis phenotypes and disease stages.

## 9 Conclusion

In summary, this review highlights m^6^A epitranscriptomic regulation as a potential integrative node linking oxidative stress, mitochondrial quality control, and programmed cell death in sepsis-induced myocardial dysfunction. By dynamically reshaping mitochondrial homeostasis and redox signal transduction, m^6^A may influence how cardiomyocytes interpret injurious stimuli and bias cell fate decisions between distinct death modalities, such as pyroptosis and ferroptosis. Conceptualizing m^6^A as a plastic regulatory hub within the redox–mitochondrial network provides a unifying, hypothesis-driven framework for understanding the heterogeneity of cardiomyocyte fate in SIMD and offers a conceptual basis for the future development of more precise and stage-adapted therapeutic strategies.

## Funding

This study was funded by the Fundamental Research Funds for Central Public Welfare Research Institutes (No. ZZ17-XRZ-028), and Guang'anmen Hospital Safeguard Program (No. GAMHH9325006).

## CRediT authorship contribution statement

**Meilian Chen:** Writing – original draft. **Binlan Fu:** Writing – original draft. **Qiaomin Wu:** Conceptualization, Visualization, Writing – review & editing.

## Declaration of competing interest

The authors declare that they have no known competing financial interests or personal relationships that could have appeared to influence the work reported in this paper.

## Data Availability

No data was used for the research described in the article.

## Abbreviations:

ALKBH5, AlkB homolog 5, RNA demethylase; AMPK, AMP-activated protein kinase; ATP, Adenosine triphosphate; BNIP3, BCL2 interacting protein 3; CASP1, Caspase-1; CLP, Cecal ligation and puncture; DRP1, Dynamin-related protein 1; FTO, Fat mass and obesity-associated protein, RNA demethylase; FUNDC1, FUN14 domain containing 1; GPX4, Glutathione peroxidase 4; GSDMD, Gasdermin D; IL-1β, Interleukin-1β; IL-6, Interleukin-6; IL-18, Interleukin-18; LC3, Microtubule-associated protein 1 light chain 3; LPS, Lipopolysaccharide; MeRIP-seq, Methylated RNA immunoprecipitation sequencing; METTL3, Methyltransferase-like 3; m6A, N6-methyladenosine; MQC, Mitochondrial quality control; mtDNA, Mitochondrial DNA; mtROS, Mitochondrial reactive oxygen species; NIX, NIP3-like protein X; NLRP3, NOD-like receptor family pyrin domain containing 3; OXPHOS, Oxidative phosphorylation; PINK1, PTEN-induced kinase 1; ROS, Reactive oxygen species; SIMD, Sepsis-induced myocardial dysfunction; SIRT3, Sirtuin 3; STAT3, Signal transducer and activator of transcription 3; TLR4, Toll-like receptor 4; TNF-α, Tumor necrosis factor alpha; WTAP, Wilms tumor 1-associating protein; YTHDF1/2/3, YTH N6-methyladenosine RNA-binding protein 1/2/3.
